# Pertussis in Southeast Asia: country-level burden and recommendations from the Global Pertussis Initiative

**DOI:** 10.1016/j.ijregi.2024.100559

**Published:** 2024-12-27

**Authors:** Phung Nguyen The Nguyen, Ulrich Heininger, Rudzani Muloiwa, Carl Heinz Wirsing von König, Daniela Hozbor, Anna Ong-Lim, Tina Q. Tan, Kevin Forsyth, Global Pertussis Initiative Southeast Asia Network

**Affiliations:** 1 University of Medicine and Pharmacy at Ho Chi Minh City, Ho Chi Minh City, Vietnam; 2 University Children's Hospital Basel, Basel, Switzerland; 3 University of Cape Town, Cape Town, South Africa; 4 Consultant, Krefeld, Germany; 5 National University of La Plata, La Plata, Argentina; 6 University of Philippines—Manila and Philippine General Hospital, Manila, Philippines; 7 Feinberg School of Medicine, Northwestern University, Chicago, United States; 8 Flinders University, Adelaide, Australia

**Keywords:** Pertussis, Vaccine, Immunization, Surveillance, Southeast Asia, Global Pertussis Initiative

## Abstract

The Global Pertussis Initiative met in March 2024 to discuss the burden of pertussis and prevention efforts in six Southeast Asian countries: Cambodia, Indonesia, Malaysia, Singapore, Thailand, and Vietnam. Except for Cambodia, pertussis is a notifiable disease; data accuracy varies within and between countries. Most cases in the region are diagnosed based on clinical suspicion. Whole-cell and acellular pertussis combination vaccines are available, but the composition differs between countries. Immunization against pertussis is well-established, but policies regarding boosting vary. Vaccination in pregnancy represents a still-neglected opportunity to protect infants. The Global Pertussis Initiative recommends enacting vaccination in pregnancy in countries without and increasing coverage in countries with policies already in place. There is a need to correct the misperception that pertussis affects children exclusively and that childhood vaccines provide lifelong protection. Education is key to widespread understanding of the threat of pertussis and removing barriers to vaccination in Southeast Asia.

## Highlights

• Education is needed to address several challenges around pertussis in Southeast Asia.
• Needs in Southeast Asia include increased surveillance and adult vaccine uptake.
• Barriers to vaccine uptake include poor awareness, access, and vaccine hesitancy.
• There is a misperception that childhood vaccines provide lifelong protection.
• There also remains a need to increase vaccination in pregnancy coverage.

## Introduction

Despite vaccine availability, pertussis (whooping cough) has been increasing worldwide [1,2] and remains one of the most poorly controlled vaccine-preventable diseases [3,4]. The Global Pertussis Initiative (GPI) was established in 2001 to raise the profile of pertussis as an important and preventable disease, improve understanding of the increasing incidence of reported pertussis, develop and recommend effective immunization strategies, and advocate for improved disease surveillance [5]. The GPI has published six sets of global- [[5], [6], [7], [8], [9], [10]] and nine sets of regional-level [5,[11], [12], [13], [14], [15], [16], [17], [18]] recommendations on managing pertussis.

As part of its ongoing efforts, the GPI met in March 2024 in Ho Chi Minh City, Vietnam to delineate the greatest challenges faced in controlling pertussis in Southeast Asia, devise potential solutions to overcome them, and discuss existing and recommended vaccine strategies.

## Surveillance, diagnosis, and epidemiologic trends

### Cambodia

In Cambodia, pertussis is not a notifiable disease and is, thus, under-recognized and under-diagnosed. Active surveillance is undertaken by the World Health Organization, with data supplied by the national surveillance program. To diagnose pertussis, a clinical case definition (Table 1) is coupled with the presence of lymphocytosis. Cases are infrequently confirmed because laboratory capacity is limited. When samples are tested, polymerase chain reaction (PCR) is used because culture and serology are not available. The number of reported pertussis cases in Cambodia has historically been low, ranging from 20 to 22 cases over 2017-2019 to <10 cases over 2020-2023 [19]. However, these low numbers are likely due to under-reporting.

**Table 1:** Overview of clinical case definitions of pertussis used, pertussis vaccine schedules, and coverage in select Southeast Asia countries.

		Cambodia	Indonesia	Malaysia	Singapore	Thailand	Vietnam
Clinical case definition		Cough lasting ≥2 weeks (or of any duration in an infant or any person in an outbreak setting without a more likely diagnosis) accompanied by ≥1 of the following: paroxysmal coughing, inspiratory whooping, post-tussive vomiting (or vomiting without other apparent cause), and/or apnea (only those aged <1 year)	Paroxysmal cough lasting ≥2 weeks accompanied by inspiratory whoop, post-tussive vomiting, or vomiting without cause; apnea ± cyanosis in those aged <1 year with cough of any duration; or clinical suspicion in a patient with cough of any duration that is not improving	Cough (no specified duration) accompanied by ≥1 of the following: paroxysms, inspiratory whooping, and/or post-tussive vomiting lacking other apparent cause	Cough accompanied by spells of repetitive (usually dry) cough, followed by sudden inspiratory effort (whoop) and post-tussive vomiting	No established definition	Afebrile; presence of whoop, nocturnal cough, or cough lasting >7 days; exposure to a confirmed case; and/or no booster dose in the previous 10 years
Primary series (recommended age for vaccination)		6, 10, and 14 weeks^a^	2, 3, and 4 months^a^	2, 3, and 5 months^b^	2, 4, and 6 months^c^	2, 4, and 6 months^d^	2, 3, and 4 months^e^
Coverage^f^	DTP1	92%	87%	99%	99%	90%	80%
	DTP3	85%	85%	97%	97%	85%	65%
Booster dose 1		No	18 months				
Booster dose 2		No	5-7 years	No	10-11 years	4-6 years	No
Booster dose 3		No	10-18 years	No	No	11-12 years	No
Adults		No	No	No	No	Every 10 years	No
Pregnant persons		No	No	Yes	Yes	Yes	Yes
Type of vaccines available		wP and aP (private sector only)	wP	aP	aP	wP and aP	

aP, acellular pertussis vaccine; DTaP, diphtheria-tetanus-acellular pertussis; DTP, diphtheria-tetanus-pertussis; DTwP, diphtheria–tetanus–whole-cell pertussis; HBV, hepatitis B virus; Hib, *Haemophilus influenzae* type b; IPV, inactivated poliovirus; wP, whole-cell pertussis vaccine.

a Infants in the public sector receive DTwP.

b Infants receive DTaP-IPV-HBV-Hib.

c Infants receive DTaP.

d Infants in the public sector receive DTwP or DTwP-HBV-Hib.

e Infants in the public sector receive DTwP-HBV-Hib.

f Coverage estimates derive from World Health Organization reports available at https://immunizationdata.who.int/dashboard.

### Indonesia

Pertussis is actively monitored in Indonesia. Epidemiologic reports are produced annually [20]. Indonesia experienced an outbreak of pertussis in 2023 that impacted 30 of 38 provinces. Overall, 2,163 cases were reported [21]—this total number of suspected pertussis cases reported between week 1 and week 46 of 2023 was 5.4-fold higher relative to the same period in 2022 (414 cases in 2022) [21]. Most affected individuals (76%) were unvaccinated, and the highest proportion of cases (38%) occurred in those aged <1 year. The outbreak is waning: suspected pertussis cases between week 1 and week 10 of 2024 was two-fold lower than that reported during the same period in 2023, with case numbers remaining elevated in only 17 of 38 provinces [20].

### Malaysia

Pertussis has been a notifiable disease in Malaysia since 1988, with fines imposed for lapses. Annual data reports are only available upon request from the Ministry of Health [22]. Cases are confirmed through isolation of *Bordetella pertussis* or the detection of bacterial DNA. PCR testing is performed only at national reference laboratories. Given the challenges in sending samples from district hospitals, especially those in East Malaysia, physicians commonly treat suspected cases based on clinical evidence alone (Table 1). This is of concern because in an analysis of 108 infants with laboratory-confirmed pertussis seen at Bintulu Hospital in East Malaysia, one-third presented with atypical symptoms [23].

Peaks in pertussis generally emerge every 4 years, with incidence rates of 3.1, 2.7, and 3.6 cases per 100,000 reported in 2015, 2019, and 2023, respectively [24]. In the intervening years, approximately one case per 100,000 was recorded [24]. The COVID-19 pandemic led to an even more marked decline in pertussis. Of the approximately 1200 cases documented in 2023, 42% occurred in those aged <5 months and 14% occurred in those aged ≥18 years [24]. With 41 pertussis-related deaths, the case fatality rate in 2023 was 3.4%. A total of 35 (85%) of these deaths occurred in infants aged <6 months [24]. Several measures were implemented to control this outbreak: providing free vaccine to all children aged <7 years, including non-citizens; treating cases and administering chemoprophylaxis to close contacts; tracing contacts; actively detecting and immunizing unvaccinated and under-vaccinated individuals; and instituting public and continuing medical education [Dr. Anand Mohan, personal communication, 2024].

### Singapore

Pertussis has been a legally notifiable disease in Singapore since 2004. The Ministry of Health releases epidemiologic reports weekly [25]. There is a mandate for clinically suspected and/or laboratory-confirmed cases to be reported within 72 hours. Information on vaccination history and, if applicable, the location of the school/childcare facility must be included, so that the Ministry of Health can monitor for the emergence of additional cases in the area over the next 2 weeks. Between 2016 and 2020, the total number of pertussis notifications ranged from 10 to 106 (annual mean of 67.8 cases) [26]. Possible explanations for the rise in cases are improved reporting/diagnosis and waning immunity among the elderly. Indeed, the first case of pertussis among those aged ≥65 years was documented in 2016; by 2018, 16% of all cases were recorded in this age group [27].

### Thailand

Pertussis is a notifiable disease in Thailand. In 2023, the Division of Epidemiology in the Department of Disease Control launched a digital surveillance platform to provide real-time data on the burden of various diseases, including pertussis. Pertussis is diagnosed based on clinical suspicion and laboratory confirmation, most commonly via PCR. Between 2017 and 2021, pertussis incidence was relatively low, with rates of 0.01-0.26 cases per 100,000 [28]. In November 2023, the incidence rate exceeded 0.5 cases per 100,000, marking the start of an outbreak that started to decline in February 2024 [29]. In 2023, 67% of reported cases occurred in those aged 0-4 years. Notably, the three provinces with the highest number of cases during the outbreak were those with the lowest vaccine coverage rates (VCRs); all three provinces are located in the deep south [29]. To control the outbreak, a three-pronged campaign was introduced in January 2024 that involved vaccinating all children aged 7 years (irrespective of vaccination history) with a pentavalent vaccine protecting against diphtheria, tetanus, and pertussis (whole cell), hepatitis B virus (HBV), and *Haemophilus influenzae* type b (Hib); pregnant individuals >16 weeks of gestation with a standalone aP vaccine (Pertagen®, approved in 2016 for those aged ≥11 years [30]); and health care providers (HCPs) caring for infants, postpartum individuals (before hospital discharge), staff at childcare centers, and household members of infants aged <6 months with Pertagen®, as part of a cocooning strategy [29].

### Vietnam

Pertussis reporting is mandatory in Vietnam; the Ministry of Health publishes reports every 2 months. Definitions of “confirmed pertussis” are not uniform across the country. Nationally, pertussis is diagnosed based on clinical suspicion (Table 1) combined with PCR, serology, or culture. Yet, at Vietnam National Children's Hospital—the largest tertiary referral children's hospital in the country—only PCR-confirmed clinical cases are considered positive.

Between 2019 and 2023, the pertussis rate in Vietnam ranged from a high of 1.4 per 100,000 to a low of 0.02 per 100,000 [31]. During this period, 77% of cases were observed in those aged <1 year, among whom 52% were unimmunized, 32% were too young to be vaccinated, and 11% were not fully immunized; only 2% of cases represented vaccine breakthroughs [31]. A total of 37 cases of pertussis were reported in 2023 (incidence of 0.04 per 100,000), all of which occurred in the comparatively marginalized northern region [31].

## Current vaccination policies

The pertussis vaccination schedule used in each country is summarized in Table 1.

### Cambodia

No catch-up campaign has been initiated in Cambodia to immunize infants who missed the recommended vaccine doses during the pandemic. Whole-cell (wP) and acellular (aP) pertussis vaccines are available for purchase in the private sector, but there are no national vaccine recommendations for older children, adolescents, adults, or specialized groups (e.g. pregnant individuals, high-risk individuals). Consequently, awareness of pertussis among non-pediatricians is low. Although there have been discussions among medical professionals in Cambodia regarding the immunization of pregnant individuals against pertussis, there is no national recommendation. The public is reportedly concerned about the effects of vaccination in pregnancy (ViP) on fetal development.

Cocooning was discussed as an additional way to protect newborns against pertussis but the need for high coverage and costs impede the likelihood of success. Moreover, due to competing health care demands, including the need to improve surveillance, pertussis is of a lower health priority relative to other infectious diseases in Cambodia. However, shifting some responsibility from mothers to family members/childcare providers would help in fostering a community approach to protecting infants; this could serve as a prelude to broader discussions regarding post-childhood vaccination.

### Indonesia

Under the National Immunization Program in Indonesia, the primary vaccination schedule involves three doses of DTwP, given at 2, 3, and 4 months of age. Booster doses of diphtheria-tetanus-pertussis (DTP) are administered at 18 months and 5-7 years, followed by one dose of either tetanus-diphtheria (Td) or tetanus-diphtheria-acellular pertussis (Tdap) among those aged 10-18 years. COVID-19 led to a marked increase in the number of missed vaccinations among children in Indonesia; to rectify this, a catch-up program involving receipt of multiple vaccines at a single health care visit and/or immunization with combination vaccines was introduced [Dr Alam, personal communication] [32]. By December 2022, the catch-up campaign had reached 61% of its target coverage [Dr Alam, personal communication] [32].

“Puskesmas” and “posyandus” are integral to the health care system in Indonesia. Puskesmas are government-run community health centers overseen by the Ministry of Health, whereas posyandus are community-based health outposts that service mothers and children in rural areas [Dr Alam, personal communication] [32]. The critical role that posyandus play in delivering health care is reflected in an app that is used to record and monitor health care services that are dispensed at anytime and anywhere. Data entered by posyandu workers (i.e. cadres) into this app are imported into the national health information platform.

Vaccination programs face challenges in Indonesia, including disparities/inequalities in coverage rates across provinces, the patriarchal nature of Indonesian society and the propensity of males to be critical of vaccines, haram-halal status (87% of citizens are Muslim), and the perception of pertussis as a mild and self-limiting disease. Vaccine refusal and hesitancy are also prevalent. In a survey of parents/caregivers of children aged <2 years undertaken in 2020, 23% of respondents refused all vaccines, and 13% considered themselves to be vaccine-hesitant [33]. Efforts to mitigate these issues include increasing immunization access, dispersing health care workers to rural areas, increasing the number of health care centers within each community, educating not only mothers [34] but also fathers on the benefits of vaccination, advising religious leaders on the halal-adherent nature of childhood vaccines, and using Geographic Information Systems to describe health conditions [Dr Alam, personal communication] [32].

### Malaysia

Vaccines in Malaysia are generally administered by nurses in the public sector and by physicians in the private sector. During the pandemic, childhood immunization rates in Malaysia declined [35]. The Malaysian Society of Infectious Diseases and Chemotherapy recommends that all pregnant individuals, irrespective of vaccination history, receive Tdap between 27 and 36 weeks of gestation during each pregnancy [36]. The importance of ViP is underscored by a hospital-based analysis that found 89% of laboratory-confirmed cases in Malaysian infants occurred in those who were too young to be fully vaccinated or who were under-vaccinated for their age [37]. Unfortunately, a 2022 survey showed that awareness of Tdap among Malaysian HCPs providing antenatal care was low (27%), with only 40% recognizing that Tdap is recommended for each pregnancy [Ismael Z, program chairman of Immunise4Life, personal communication]. In 2024, Malaysia plans to provide Tdap to all pregnant individuals, including non-citizens, for free and to push the offering of Tdap up from the third to the second trimester [Dr Anand Mohan, personal communication, 2024]. Another challenge facing pertussis control efforts is health care access; in East Malaysia, the nearest health clinic may be hours away or accessible only by water or air.

### Singapore

Coverage rates for the first (93%) and second (94%) boosters in Singapore are high [38]. These high VCRs are attributable to universal vaccination requirements and recommendations for all Singaporeans and permanent/non-permanent residents, vaccination requirements for children attending primary school, provision of free vaccines to Singaporeans and permanent residents and of subsidized vaccines to non-permanent residents, a high level of health care access, and a lack of religious exemptions. However, COVID-19 reduced the childhood vaccination rates across Singapore [39].

Since 2017, obstetricians/gynecologists have worked to administer Tdap to pregnant individuals, generally between 16 and 32 weeks of gestation. In a survey of 70 Singaporean obstetricians performed in 2017, 83% considered Tdap to be safe and effective in pregnant individuals [40]. Barriers to obstetrician administration of Tdap included lack of time to educate patients (41%), uncertainty over vaccine recommendations (34%), and a belief that vaccination was unnecessary because pertussis was not a major problem locally (27%) [40]. Providing patients with educational materials on the benefits of vaccination before the clinic visit, such as via text message, is one potential strategy to overcome this.

The Singapore National Adult Immunization Schedule has recommended since 2017 that adults with certain medical conditions, those with no history of previous vaccination, and those vaccinated >10 years ago receive Tdap [41]. Given the increased burden of pertussis among those aged ≥65 years, there is a movement for general practitioners and geriatricians to vaccinate patients against pertussis every 10 years. As additional incentivization, some insurance companies offer discounts on premiums to members who provide proof of decennial vaccination with Tdap.

There is a need to improve public awareness of pertussis and the benefits of vaccination. Barriers to adult vaccination include the misperception that vaccination is not part of healthy aging, lack of clarity on who should administer Tdap (e.g. general practitioner, specialist), absence of regular touchpoints with HCPs, and the fact that pertussis is not seasonal, meaning that there is no obvious time to enact reminder campaigns [27]. Cocooning was considered unfeasible in Singapore, given the high rates of flux into and out of the country.

### Thailand

Approximately 85% of Thai children are immunized with a whole-cell vaccine under the Expanded Program on Immunization (EPI); the remainder receive an acellular formulation (e.g. DTaP-IPV-HBV-Hib, DTaP-IPV-Hib, DTaP-IPV) in the private sector. Coverage rates for the first and second boosters are 84% and 74%, respectively [42]. Additional boosting is recommended for persons aged 11-12 years, followed by every 10 years thereafter; however, Tdap is only available (for purchase) through the private sector, whereas under the EPI, Td (without the pertussis component) is administered. Overall, COVID-19 had little impact on immunization rates for the primary series but negatively affected booster uptake, particularly, in the deep south [42].

As the Thai population is aging, immunizing adults against pertussis is becoming increasingly important. The Ministry of Health recommends that individuals who are pregnant receive an acellular vaccine—either Tdap or standalone aP—at 27-36 weeks gestation for each pregnancy. However, only 2% of pregnant individuals were estimated to have received it in 2023 and 8% in 2024 [42]. Those who receive standalone aP must also receive Td, if indicated; the cost of these two vaccines is currently less than that of Tdap.

### Vietnam

Approximately 85% of all vaccines in Vietnam are administered by the EPI; the remainder is procured in the private sector, where aP vaccines are available for purchase. Due to the pandemic, Vietnam experienced a vaccine shortage in 2021-2023. In 2023, the coverage rate for DTP3 was only 65% [32]. A catch-up campaign is not planned; however, in 2024, vaccination against pertussis became a requisite for school entry in 50% of provinces, with a nationwide requirement planned for 2025.

Although Tdap is approved for use in adolescents and pregnant individuals, uptake is poor due to, in part, low public awareness and out-of-pocket costs. There is a need to educate physicians who see adolescents and adults about the ongoing dangers of pertussis. To improve VCRs among those who are pregnant, the meeting attendees suggested strengthening coordination between the EPI and obstetricians/pediatricians and enhancing communication with patients before and during pregnancy.

## Discussion

### Surveillance, diagnosis, and epidemiology

Pertussis case reporting is mandatory in all represented countries, except for Cambodia. However, data accuracy varies within and between countries, with poorer quality reporting in remote areas. Cost and the perception that pertussis does not pose a substantial clinical burden are obstacles to active surveillance. Yet, based on the experiences of the GPI, if efforts are made to look for pertussis, it will be found. Although infants appear to be most commonly affected, seroprevalence studies would help to delineate the true burden of *B. pertussis* infections.

Most cases of pertussis in the region are diagnosed (and treated) based on clinical suspicion due to the time lag and costs associated with laboratory confirmation. Clinical case definitions of pertussis vary among the six Southeast Asian countries (Table 1). Exclusive use of clinical case definitions is of concern because pertussis symptoms vary by vaccination status and age. Moreover, compared with pediatricians, awareness of pertussis is generally poorer for HCPs specializing in the care of older individuals, impeding the identification of suspected clinical cases in adults. Poor recognition of pertussis in adolescents/adults is a driver of disease transmission among infants and children [5,7,9].

Delays in laboratory diagnosis are attributable to the limited number of testing facilities, with many health care institutions sending samples to regional or national reference laboratories, and to the use of batch testing. Overall, PCR is the most frequent method of laboratory confirmation used in the region, with culture infrequently used. As with surveillance, cost is a major barrier to clinical sample testing.

### Vaccination availability, scheduling, and coverage

Only wP vaccines are available in Indonesia, whereas only aP vaccines are available in Malaysia and Singapore. Although both vaccine types are licensed for use in Cambodia, Thailand, and Vietnam, access to acellular formulations is limited to the private sector. Immunization against pertussis is well-established in each country, but booster dosing practices vary (Table 1). Generally, the coverage rates for the primary series are good (≥85%), but national estimates obscure local variations, such as in remote areas where uptake is lower (e.g. northern region of Vietnam).

Recommendations are in place for pregnant persons to receive Tdap in Malaysia, Singapore, Thailand (Tdap or standalone aP), and Vietnam (Table 1), but coverage is low. There are no national recommendations for ViP in Cambodia or Indonesia. However, even in the absence of national policies for pregnant persons, conversations can start in the private sector, admittedly in those who are more affluent. Over time, the experience with pertussis immunization in the private sector would grow, such that if a policy were enacted at the national level, the practice will have already become “normalized,” lowering the likelihood of pushback in the general population.

Barriers to vaccine uptake in the region include competing priorities, monetary constraints, poor awareness of pertussis and immunization policies, insufficient health care access, family dynamics (i.e. who is making decisions regarding immunizations), and hesitancy fueled by vocal minorities (e.g. anti-vaxxers). Strategies to increase vaccine uptake are outlined in Table 2. Although little can be done in the way of reprioritizing pertussis in the face of more pressing healthcare issues because other diseases become less of a concern, the infrastructure developed can be applied to the management and control of pertussis at the appropriate time. As to monetary constraints, international support (e.g. GAVI) is needed for governments of low-to-middle-income countries to purchase pertussis vaccines. Because a vaccine is obviously only effective if administered, government subsidies are needed to reduce or eliminate out-of-pocket costs for recipients. Yet, individuals must be willing to be immunized against pertussis. It is in this arena that public education and outreach are key. Emphasizing the history of use of wP and aP vaccines represents one strategy. The legacy of these vaccines may serve to increase confidence in and, ultimately, acceptance of the need for lifelong protection against pertussis. Health authorities can consider showcasing how vaccination campaigns led to the control of recent pertussis outbreaks in Indonesia, Malaysia, and Thailand. Mass media campaigns depicting individuals personally affected by pertussis will also increase awareness. These personal stories can illustrate how infants are vulnerable to pertussis and how the immunization of mothers and other household contacts can protect infants until they themselves are old enough to be vaccinated. In countries such as Vietnam, parents purchase childhood vaccines in advance. If it were possible to frame ViP (and the passive immunity conferred) as the infant's “first” pertussis vaccine, this may help to increase uptake and protect the most vulnerable of age groups. Countries can also look to the processes implemented for immunizations that are now considered standard during pregnancy (e.g. tetanus, diphtheria). In addition, transitioning from use of Td to Tdap would increase coverage rates among those who are pregnant. To maximize uptake of ViP, education campaigns should be undertaken such that expectant mothers request and obstetricians/gynecologists/midwives recommend Tdap.

**Table 2:** Strategies to increase pertussis immunization rates in Southeast Asia.

**Structural** • Leverage infrastructure erected for other infectious diseases (e.g. COVID-19, polio, tetanus) • Establish health outposts/traveling clinics and engage local leaders to reach the residents of remote areas • Set up “vaccination days” at the workplace to increase accessibility for adults **Financial** • Procure international financial support to enable low-to-middle–income governments to purchase pertussis vaccines • Introduce government subsidies to minimize or eliminate out-of-pocket costs for vaccine recipients **Policy** • Consider mandating pertussis vaccination for school entry • Establish national vaccination policies for pregnant persons and household contacts of infants **Public outreach** • Emphasize the history of use of pertussis vaccines to increase public confidence and trust in vaccines • Educate on how vaccination campaigns have led to the control of recent pertussis outbreaks • Develop mass media campaigns highlighting those personally affected by pertussis • Have trusted health authorities establish a social media presence to create disease awareness, mitigate vaccine fears, and counteract the efforts of anti-vaccination groups • Send text alerts to remind individuals of immunization needs **Healthcare professionals** • Develop continuing medical education courses that address how childhood vaccines do not provide lifelong protection against pertussis and that emphasize the benefits of and need for booster doses • Modify existing medical curricula such that future healthcare professionals understand the need to protect against pertussis beyond infancy/childhood **Persons who are pregnant** • Elevate the importance of ViP • Increase awareness of pertussis and access to Tdap during routine prenatal visits • Frame ViP as representative of the infant's “first” pertussis vaccine • Normalize pertussis immunization during pregnancy, as was done for tetanus • Switch from use of Td to Tdap

Td, tetanus-diphtheria vaccine; Tdap, tetanus-diphtheria-acellular pertussis vaccine; ViP, vaccination in pregnancy.

Given their ability to transmit *B. pertussis* to unimmunized or partially immunized newborns, all non-pregnant adults should receive Tdap. However, there are challenges associated with vaccinating this age group, including poor recognition of pertussis in adults, inadequate disease surveillance, misperceptions that pertussis only affects children and that childhood vaccines provide lifelong protection, absence of national-level vaccine recommendations, and lack of understanding over how vaccinating adults can protect unimmunized or under-immunized infants.

Other than for pediatricians, vaccination is not typically a focus of medical curricula; however, it is critical that providers of health care to adults, such as general practitioners, obstetricians/gynecologists/midwives, and geriatricians, understand the need to protect against pertussis beyond infancy/childhood. Thus, the GPI recommends (where feasible) engaging professional societies to design continuing medical courses focused on immunization and ensure that the importance of vaccination throughout life is stressed during the training of future HCPs.

Other ways to increase vaccine coverage include mandating pertussis vaccination for school entry; establishing health outposts/traveling clinics; engaging local leaders to reach residents of remote areas; setting up “vaccination days” at the workplace; sending text alerts to remind individuals of immunization needs; and having trusted health authorities establish a social media presence to create disease awareness, mitigate vaccine fears, and counteract the efforts of anti-vaccination groups (Table 2). The immunization recommendations put forth by respected health agencies, such as the Strategic Advisory Group of Experts on Immunization of the World Health Organization, may influence governments to implement similar strategies. However, the Strategic Advisory Group of Experts on Immunization policy on pertussis vaccination has not been updated since 2014 [43].

### Impact of COVID-19

One positive consequence of the COVID-19 pandemic was the scaling up of diagnostic capabilities. However, the decline in COVID-19 may lead to the closure of testing facilities and a reversion to pre-pandemic laboratory access. During the height of the pandemic, reported cases of pertussis (and other respiratory illnesses) declined across the six Southeast Asian countries but so did immunization rates. Pertussis cases have returned to pre-pandemic levels in most countries; indeed, Indonesia, Malaysia, Thailand, and Vietnam experienced outbreaks in 2023-2024. These outbreaks may reflect not only lapses in vaccination coverage but also cycles of endemicity. Vaccination campaigns were implemented to control these outbreaks.

Unfortunately, owing to a pandemic-related vaccine shortage, immunization rates for the primary series and 18-month booster in Vietnam are currently subpar. Reductions in VCRs trigger outbreaks, which the public may erroneously attribute to a lack of vaccine effectiveness. Although COVID-19 disrupted health care delivery and vaccine availability, innovations developed in response to the pandemic did lead to improvements in resourcing, health care access, and communication that could potentially be leveraged for other infectious diseases, including pertussis.

## Conclusion

Several challenges to managing pertussis in Southeast Asia were identified, including the paucity of robust epidemiologic data and need to enhance disease surveillance, laboratory diagnosis, and vaccine uptake among adults. Improvements in resourcing—money and infrastructure—are needed to enhance disease surveillance and the number of laboratory-confirmed cases. This is critical because (1) it is difficult to obtain accurate epidemiologic data in the absence of robust surveillance and (2) it is the epidemiology of an infectious disease that informs vaccination policies. Although vaccination rates vary across the six Southeast Asian countries, overall coverage for DTP3 generally exceeds 85%. Because national estimates obscure local variations, it is critical to continue efforts to expand (and sustain) coverage to all regions within individual countries. ViP represents a still-neglected opportunity to protect infants who are too young to have initiated or completed the primary series. Consequently, efforts are needed to increase ViP coverage throughout the region and to improve education and awareness about pertussis in the public sector and among HCPs who care for pregnant persons. Although the tools—surveillance, laboratory diagnosis, and vaccines—are available, augmenting access to them and fostering understanding of the benefits of post-childhood immunization across all population strata (e.g. public, medical professionals, policymakers) will help to curtail the burden that pertussis poses to Southeast Asia.

## Declarations of competing interest

The authors are scientific experts and, as such, received honoraria for their participation in this live meeting from Sanofi. In addition, U.H. was a member of the Collaboration of European Experts on Pertussis Awareness Generation (CEEPAG; he was a group member until 2021) and has received honoraria for participation in previously associated live meetings from Sanofi, USA, and Sanofi, France, respectively, as well as receiving honoraria for educational activities from GSK, Pfizer, MSD, Sanofi, and InfectoPharm. R.M. has received honoraria for educational activities from MSD and Sanofi. C.H.W.K. has received honoraria for attending meetings sponsored by Sanofi, GSK Biologicals SA, MSD, and Novartis Vaccines. T.Q.T. has received grants from Merck and Sanofi, personal fees from GSK Biologicals and Sanofi, and honoraria from Sanofi. K.F. has also previously received honoraria from Sanofi. The authors declare no other conflict of interest.
